# Medication adherence and persistence in the treatment of Canadian ulcerative colitis patients: analyses with the RAMQ database

**DOI:** 10.1186/1471-230X-13-23

**Published:** 2013-01-30

**Authors:** Jean Lachaine, Linnette Yen, Catherine Beauchemin, Paul Hodgkins

**Affiliations:** 1 Faculty of Pharmacy, University of Montreal, PO Box 6128, Station Centre-ville, Montreal, Quebec, H3C 3J7, Canada; 2 Global Health Economics and Outcomes Research, Shire Development LLC, Wayne, PA, USA

**Keywords:** Ulcerative colitis, Anti-inflammatory drugs, Adherence, Persistence

## Abstract

**Background:**

Although high non-adherence to medication has been noticed for ulcerative colitis (UC), little is known about adherence to mesalamine treatments and determinants that can predict adherence. The objective of this study was to assess adherence and persistence to mesalamine treatments and their potential determinants in mild to moderate UC patients in a real-life setting in Quebec, Canada.

**Methods:**

A retrospective prescription and medical claims analysis was conducted using a random sample of mesalamine users with UC. For inclusion, patients were required to initiate an oral mesalamine treatment between January 2005 and December 2009. Patients with a diagnosis of Crohn’s disease were excluded. Treatment adherence (medication possession ratio [MPR]) and persistence were evaluated over a 1-year period after the index prescription using the Kaplan-Meier method with log-rank test and stepwise regression to identify potential determinants.

**Results:**

A sample of 1,681 of the new oral mesalamine users (mean age = 55.3) patients was obtained. Overall, the percentage of patients with a MPR of 80% or greater at 12 months was 27.7%, while persistence was 45.5%. Among patients treated with mesalamine delayed/extended-release tablets (Mezavant®), adherence and persistence were 40.9% and 71.9%, respectively. Predictors of high adherence included, male gender (OR=1.3; 95% confidence interval [CI]=1.1–1.6), older age (>60 years; OR=1.6; 95% CI=1.3–2.0) and current use of corticosteroids (OR=1.4; 95% CI=1.1–1.8). Predictors of high persistence included male sex (OR=1.4; 95% CI=1.1–1.7), current use of corticosteroids (OR=1.4; 95% CI=1.1–1.7) and presence of hypertension or respiratory diseases (OR=1.2; 95% CI=1.01–1.55).

**Conclusions:**

The majority of patients with UC exhibited low adherence and persistence to mesalamine treatments. Various determinants of improved adherence and persistence were identified.

## Background

Ulcerative colitis (UC) is a ubiquitous disease, with a worldwide incidence rate varying from 0.5 to 24.5/100,000 persons per year [1,2]. The highest incidence rates mainly occur in the most developed regions, notably North America and Western Europe. In Canada, the prevalence and incidence of UC are estimated as 267 cases per 100,000 and 12.9 cases per 100,000, respectively [3]. In the United States, UC prevalence and incidence rates are estimated as 214 per 100,000 and 8.8 cases per 100,000 [4]. From an European perspective, in 2000, the United Kingdom had a UC prevalence and incidence rate of 153 cases per 100,000 and 19 cases per 100,000, respectively [5].

Substantial costs are associated with the management of the disease. According to Cohen et al., the 2008 overall cost of UC in the European Union varied between €12.5–29.1 billion, which comprised medical costs (€5.4–12.6 billion) and absenteeism associated costs (€7.1–16.5 billion) [6]. Consistent with these figures, the economic burden associated with UC in the United States was estimated at $US 8.1–14.9 billion in 2008. Health care costs represented $US 3.4–8.6 billion while productivity losses were $US 4.7–6.3 billion [6].

Still incompletely understood, UC is an inflammatory bowel disease that results from an excessive immune response leading to intestinal inflammation [1,7]. The course of UC over time may consist of a repeated state of symptom exacerbation punctuated with periods of remission [8]. The symptomatic relapse can be associated with constant diarrhea, rectal bleeding, abdominal pain, weight loss, and fatigue [9]. A Westernized environment and lifestyle are linked to the appearance of inflammatory bowel disease, which may be associated with smoking, diets high in fat and sugar, medication use, stress, geographic localization and high socioeconomic status [10]. However, their role remains uncertain and unconfirmed [1,7,11]. The only known risk factor associated with UC is heredity [8,12]. Other factors, such as smoking status, have been associated with UC [8,11].

The American College of Gastroenterology Clinical Practice Guidelines classify UC into 4 disease severities: (1) mild disease, (2) moderate disease (>4 stools/day, minimal signs of toxicity), (3) severe disease (>6 stools/day, fever, tachycardia, anemia, elevated erythrocyte sedimentation rate), and (4) fulminant disease (>10 stools/day, continuous bleeding, toxicity, abdominal tenderness and distension, transfusion requirement, colonic dilation) [13]. Since there is no curative treatment for UC, this inflammatory disorder is generally controlled with pharmacological agents that are either used to control the symptoms and/or to attenuate the inflammation. Currently, the anti-inflammatory first line therapies for mild to moderate UC, include olsalazine and 5-aminosalicylic acid (5-ASA) also known as mesalamine [14]. In Canada, the most commonly used treatments of the active principle 5-ASA are commercialized under 4 different names: Mezavant®, Asacol® and generics, Salofalk®, and Pentasa® [15]. Compared to the other 5-ASA treatments, which need to be taken as 2 to 4 times per day, Mezavant® was designed to allow delayed and gradual release of 5-ASA in the colon due to its Multi Matrix® (MMX®) hydrophilic and lipophilic coating, thus it can be taken only once daily and was shown in clinical trails to be as effective as twice daily mesalamine formulations [16,17].

In all chronic diseases, treatment adherence and persistence remain significant concerns, as these conditions may require medications to be taken for the entire or a major part of the lifespan. Consequences of non-adherence and persistence can be many including relapsing or worsening of disease, increased morbidity, or even mortality. Indeed, Kane suggested that the major consequences of non-adherence to 5-ASA for patients with UC are a five-fold higher risk of relapse, an increased risk of colorectal cancer and a reduced quality of life [18]. Identification of risk factors associated with non-adherence in UC may improve patient management and consequently reduce relapse episodes, by providing more effective interventions intended for improving adherence. Importantly, the lack of adherence to UC treatments has been associated with a significant increase of costs, as non-adherent patients who are at an increased risk of relapse are likely to contribute to the overall high costs associated with the treatment of UC [19].

Although high non-adherence to medication has been reported in mild to moderate UC, little is known about adherence to the different 5-ASA treatments and determinants that can predict adherence and persistence. Therefore, the objectives of the present study were to estimate adherence and persistence to 5-ASA treatments and to identify the potential determinants of adherence and persistence in a real-life setting.

## Methods

### Study database and population

A retrospective prescription claims study was conducted using data from pharmaceutical services of the Quebec provincial health plan database (Régie de l’assurance maladie du Québec, RAMQ). As with other Canadian provinces, Quebec has a universal health care program fully subsidized by the provincial government, covering physician services and hospitalization for the entire population. This health care program is complemented by a public drug plan provided to a significant proportion of the population. The provincial drug reimbursement program is not universal. It covers persons aged ≥65 years, beneficiaries of the social assistance program, and individuals who do not have access to a private plan of medication insurance. Although, the database comprises patients from all age groups, the persons aged <65 years are underrepresented. In 2009, approximately 3.2 million persons (40% of the total population) were covered by the provincial drug reimbursement program. Depending on their incomes, some of the beneficiaries of the drug plan co-pay or pay part of their premiums.

The RAMQ pharmaceutical services database contains information from pharmacy claims for dispensed medications reimbursed by the program, but not for medications received in a hospital. In addition, data obtained from the RAMQ comprise an encrypted patient identifier, which enabled linkage of individual patient information while preserving anonymity. The RAMQ data are anonymized and are publicly available, at a cost, for research purposes. The RAMQ Direction for analysis and management of information manage the distribution of RAMQ data to external parties.

Data on prescription claims were obtained from the RAMQ database for the period from January 1, 2004 to December 31, 2009 for subjects who did not have a diagnosis of Crohn’s disease (the International Classification of Diseases, Ninth Revision [ICD-9] diagnostic codes 555.0–555.9) and who had received ≥1 of the 5-ASA treatments during the study period. New users (operationalized as no prescription claim in the 3 months prior to the date of the first prescription fill [index date]) of a mesalamine treatment (Asacol® or generics, Pentasa®, Salofalk®, or Mezavant®) were eligible for inclusion in the analysis. Treatment adherence (based on medication possession ratio [MPR]) and persistence were evaluated over a 1-year period after the index prescription.

### Data analysis

Characteristics of the study population were analyzed in terms of age, sex, and geographical localization. Comorbidity level was also estimated by calculating a chronic disease score based on the Von Korff method [20]. Number and severity of comorbidities were assessed according to the patients’ medication profile. More specifically, occurrences of selected medication utilization during the study period were used to calculate the chronic disease score. Finally, the rate of predefined most common comorbidities (heart disease, respiratory illness, hypertension, and diabetes) was evaluated for the entire study population.

Descriptive statistics were generated on treatment characteristics for each 5-ASA treatment including the number and proportion of users, the number and average duration of prescriptions, and the average quantity of tablets per prescription.

Adherence and persistence were evaluated in new users with no prescription claim in the 3 months prior to the date of first prescription fill. Adherence was defined as the consistency of medication consumption as detailed in the recommended treatment regimen while persistence was defined as the long-term continuation of treatment.

Adherence to treatment was estimated by calculating the number of days for which medication was received divided by the expected duration of the treatment (MPR) [21]. Treatment adherence was estimated over a 1-year period [21]. The proportion of adherent patients was estimated for each 5-ASA treatment. Patients were considered adherent to their 5-ASA medication if their medication possession ratio was ≥80% for a 5-ASA treatment; a criterion used in other UC-adherence studies [21,22]. As a complementary analysis, the proportion of patients with an MPR of ≥50% was also calculated.

Treatment persistence was estimated over a 1-year period, following the index prescription. A patient was considered persistent for as long as the 5-ASA treatment was not definitely ceased. Treatment cessation was determined if the patient did not receive a 5-ASA treatment for a period of ≥60 days, representing twice the usual refill time. Patients who switched to another treatment of mesalamine were considered non-persistent to the initial medication. Time to discontinuation was calculated and compared using the Kaplan-Meier method with log-rank test.

In order to determine which factors can have an impact on adherence and persistence to 5-ASA treatments, different covariates were considered in 2 independent models (1 for patient demographics and 1 for comorbidities). Potential determinants for adherence and persistence comprised sex, age, comorbidities, and prior or current use of corticosteroids. Relative adherence and persistence were estimated in consideration of each of these potential determinants: patient age was dichotomized into 2 cohorts (those <60 years and those ≥60 years); comorbidities included were heart disease, respiratory illness, hypertension, and diabetes: chronic disease score was also dichotomized (those with a score less than the median value of 6 and those with a score of ≥6). Finally, patients who received a corticosteroid in the year prior to the initiation of the 5-ASA treatment were compared to those with no previous use of corticosteroids, as well as patients who were currently using a corticosteroid were compared to those that were not currently using corticosteroids. Stepwise backward regression modeling was employed to identify potential determinants for adherence and persistence from available data on the study cohort. Logistic regression analysis was performed to calculate odds ratios (ORs) and 95% confidence intervals (CIs).

For statistical analyses of comparisons between 2 groups, means of continuous variables were compared using Student’s t test, and for comparisons among several groups, the F-test with one-way analysis of variance (ANOVA) was conducted. Statistical analyses were performed for comparisons between 5-ASA treatments in terms of treatment characteristics, adherence and persistence by performing the chi-square test. Statistical significance was defined as *P* <0.05.

## Results

### Study sample

**Figure 1:**
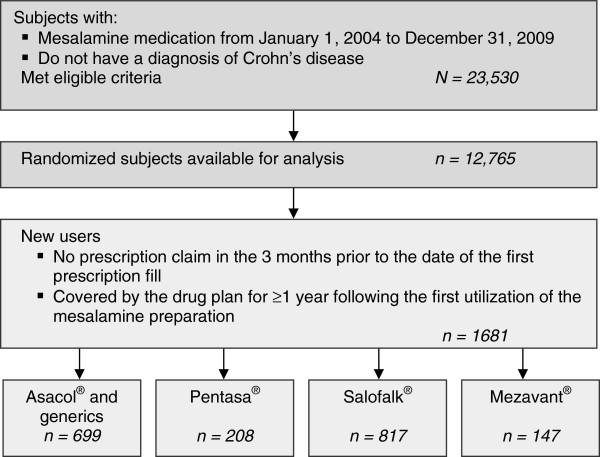
Sample selection and attrition.

During the period from January 1, 2004 to December 31, 2009, a total of 23,530 patients, covered by the provincial drug plan during that period, received ≥1prescription for a mesalamine treatment. In accordance with the RAMQ’s restrictions on the number of subjects available for analysis by external parties, data were obtained for a random sample of 12,765 patients. Of these, 1681 were new users of an oral mesalamine preparation, who did not have a diagnosis of Crohn’s disease and who were covered by the drug plan for ≥1 year following the first utilization of the mesalamine preparation (Figure 1). The mean age of the study population was estimated at 55.3 years (standard deviation = 17.8), with a slightly higher proportion of females (56.6% vs 43.4% for males). The majority of the sample was aged between 60 to 79 years (43.7%), while 74.4% of the sample was comprised of patients aged between 40 to 79 years. All defined comorbidities (hypertension, heart disease, respiratory illness, and diabetes) presented a high rate in this group, with proportions ranging from 32.7% for diabetes to 50.1% for hypertension (Table 1). The 1681 new oral 5-ASA users received a total of 17,062 scripts for mesalamine treatments (Table 2). The average number of scripts per patient was 8.6 for Asacol® and generics, 7.8 for Pentasa®, 9.0 for Salofalk®, and 14.4 for Mezavant®. The average duration (in days) per script was 24.0 for Asacol® and generics, 19.8 for Pentasa®, 23.0 for Salofalk®, and 27.2 for Mezavant®. Based on number of scripts, Salofalk® was the most commonly prescribed and Pentasa® was the least prescribed treatment.

**Table 1:** Study population

	**n = 1681**
**Age group, y, n (%)**	
· <20	60 (3.6)
· 20–39	287 (17.0)
· 40–59	516 (30.7)
· 60–79	734 (43.7)
· 80+	84 (5.0)
Mean age (SD)	55.3 (17.8)
**Male/Female, n (%)**	729 (43.4)/952(56.6)
**Rate of comorbidities, n (%)**	
· Hypertension	842 (50.1)
· Heart disease	695 (41.3)
· Respiratory illness	570 (33.9)
· Diabetes	549 (32.7)

SD, standard deviation.

**Table 2:** Characteristics of the prescriptions in the first year after index date

**Treatment (no. of users)**	**No. of scripts**	**No. of scripts per patient**	**Average quantity per script (SD)**	**Average duration per script in days (SD)**	**Median duration per script in days**
Asacol® and generics (699)	5994	8.6	146 (102)	24.0 (12.8)	30
Pentasa® (208)	1627	7.8	111.7 (92.9)	19.8 (12.7)	30
Salofalk® (817)	7317	9.0	140.0 (96.8)	23.0 (14.1)	30
Mezavant® (147)	2124	14.4	77.1 (40.5)	27.2 (11.7)	30

SD, standard deviation.

### Patient adherence and persistence to 5-ASA treatments

**Figure 2:**
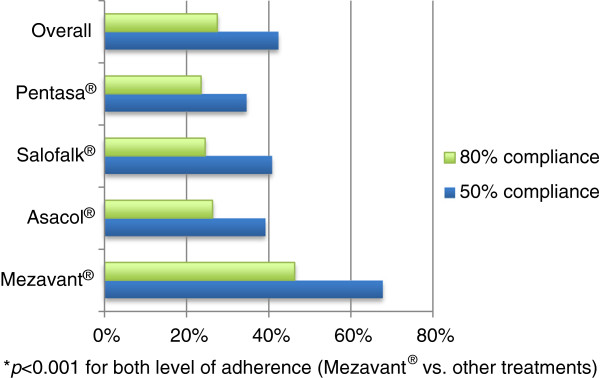
Adherence to 5-ASA Treatments.

**Figure 3:**
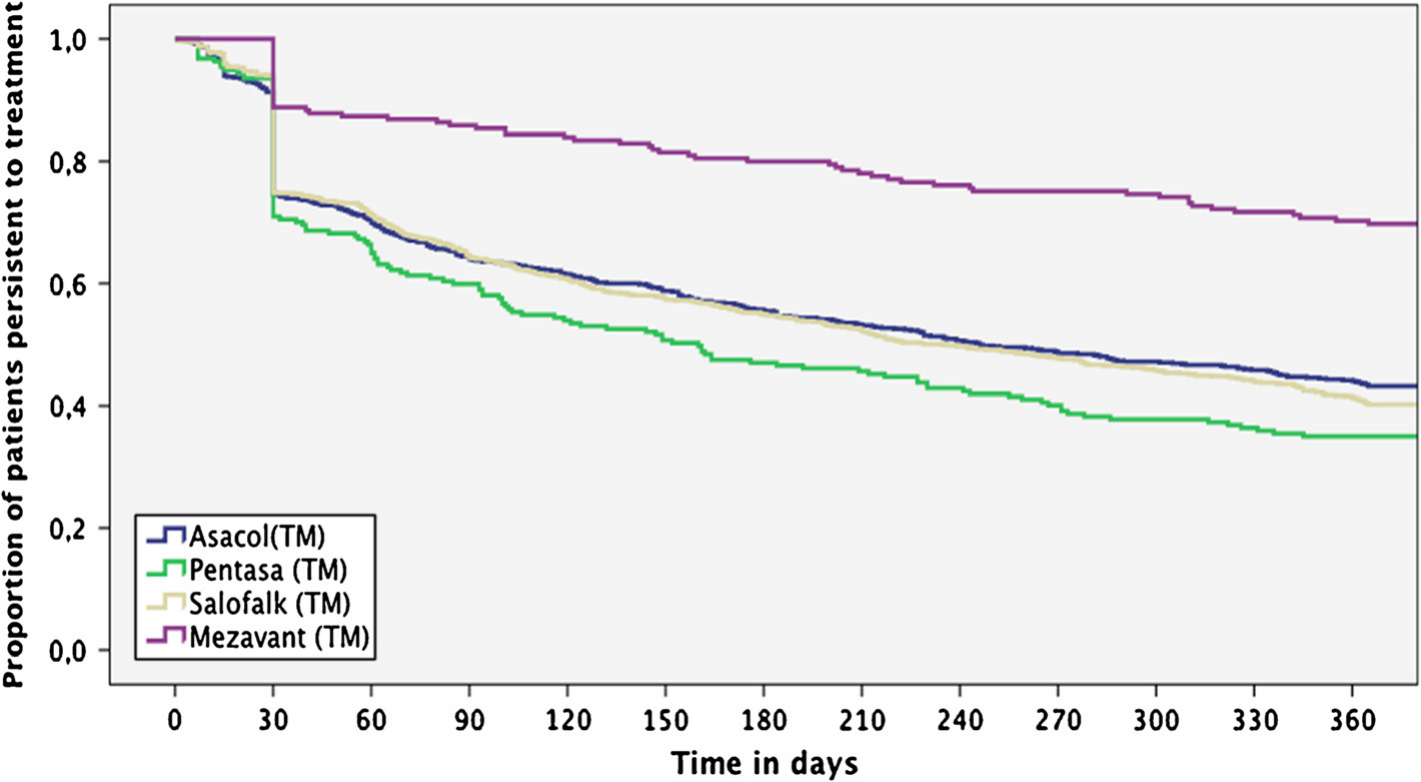
Persistence to 5-ASA treatments.

Treatment patterns with oral 5-ASA treatments were evaluated for the study population in terms of treatment adherence and persistence. Treatment adherence was reported using a threshold of both 50% and 80% adherence (Figure 2). In general, treatment adherence over 12 months was relatively poor, with an overall 80% adherence to 5-ASA treatments of 27.7% and a 50% adherence of 42.4%. However, adherence was significantly higher with Mezavant® users than with any other oral treatments, with 40.9% of adherent patients compared to ranging from 26.4% for Pentasa® to 28.5% for Asacol® and generics; *P* <0.001 for Mezavant® versus other treatments (Figure 2). Treatment persistence was estimated over a 1-year period, following the index prescription. As shown in Figure 3, treatment persistence decreased with time, although the proportion of patients who were persistent during the 1-year period on Mezavant® was significantly higher when compared with those on any other 5-ASA treatment (71.9% vs 42.8% for Pentasa® to 47.5% for Asacol® and generics; *P* <0.001). Overall persistence to 5-ASA treatments at 12 months was 45.5% (Figure 3).

### Determinants of adherence and persistence

Impact of potential determinants of adherence and persistence were evaluated. These include sex, age, current or prior use of corticosteroids, as well as the impact of having a diagnosis of another chronic disease and chronic disease score.

Results from the regression models indicated that male sex, older age (>60), and current use of corticosteroids were associated with higher adherence to mesalamine treatments (OR [95% CI] = 1.3 [1.05–1.6], 1.6 [1.3–2.0], and 1.4 [1.1–1.8], respectively). No other covariates were found to be associated with improved adherence. Male sex, current use of corticosteroids, and presence of hypertension or respiratory disease were associated with higher persistence (OR [95% CI] = 1.4 [1.1–1.7], 1.4 [1.1–1.7], 1.2 [1.01–1.50], and 1.2 [1.004–1.548] respectively; Table 3).

**Table 3:** Impact of patients’ characteristics and comorbidities on adherence and persistence to UC treatments

	**Adherence**		**Persistence**	
	**Patient’s adherent over 12 months n (%)**	**Adjusted OR [95% CI]**	**Patient’s persistent at 12 months n (%)**	**Adjusted OR [95% CI]**
Sex				
· Male	222 (30.5)	1.3	365 (50.1)	1.4
· Female	243 (25.5)	[1.05–1.6]	400 (42.0)	[1.1–1.7]
Age				
· < 60	201 (23.3)	1.6	381 (44.1)	NS
· ≥ 60	264 (32.3)	[1.3–2.0]	384 (49.9)	
Previous use of corticosteroids				
· Yes	142 (28.2)	NS	231 (45.8)	NS
· No	323 (27.4)		534 (45.4)	
Concurrent use of corticosteroids				
· Yes	179 (32.7)	1.4	273 (49.8)	1.4
· No	286 (25.2)	[1.1–1.8]	492 (43.4)	[1.1–1.7]
Heart disease				
· Yes	210 (30.2)	NS	321 (46.2)	NS
· No	255 (25.9)		444 (45.0)	
Respiratory illness				
· Yes	153 (26.8)	NS	247 (43.3)	1.2
· No	312 (28.1)		518 (46.6)	[1.004–1.548]
Hypertension				
· Yes	262 (31.1)	NS	403 (47.9)	1.2
· No	203 (24.2)		362 (43.1)	[1.01–1.50]
Diabetes				
· Yes	167 (30.4)	NS	515 (45.5)	NS
· No	298 (26.3)		250 (45.5)	
Chronic disease score				
· Yes	159 (23.2)	NS	296 (43.3)	NS
· No	306 (30.7)		469 (47.0)	

CI, confidence interval; NS, not significant; OR, odds ratio; UC, ulcerative colitis.

## Discussion

Our results demonstrated that, in general, adherence and persistence observed with 5-ASA therapy in this sample were poor. Indeed, other studies reported that adherence to 5-ASA therapy is low, as many as 60% of patients failing to adhere to a prescribed dose regimen and taking <70% of their prescribed medication [23,24]. Also, for inflammatory bowel disease, treatment non-adherence varies between 30 and 43% and was estimated at 42% for UC patients receiving first-line therapies [25,26]. The persistence at 12 months to 5-ASA therapy was previously reported at 55% [27]. The poor adherence and persistence observed can be explained by different factors specifically related to UC and the associated treatment regimens. Even if the 5-ASA therapy maintenance is effective in reducing the number of UC relapses, adherence rates are particularly poor among patients in symptomatic remission, as some of these patients are unable to see the need for medication during the periods of symptom quiescence [24,28]. Lack of medical supervision, concomitant medications, side effects and cost of prescriptions were frequent reasons for treatment non-compliance cited by patients in a North American study [18]. However, the most cited factor was the inconvenient dosage schedule of 5-ASA medications, which required a large number of tablets per day. Further, in another US study of 99 patients with quiescent UC, when asked their reasons for non-adherence to their prescribed regimen, 30% of patients who responded said there were too many pills, 20% did not think they needed that much medication, and 50% said they forgot [28]. A recent study evaluating efficacy and adherence of once daily versus 3 times daily dosing of Asacol® showed that once daily dosing led to reduced relapse rate, mainly due to improved adherence [3]. Consistent with these observations, we observed significantly better adherence and persistence with Mezavant®, which is a once daily oral treatment. As 5-ASA non-adherence is a major factor in recurrence of UC, a once daily oral treatment with an extended drug release could be a therapeutic option to improve adherence in UC patients and then to maintain therapy leading to better outcomes and to prevent relapses and complications.

This study also evaluated determinants of adherence in terms of patient characteristics and concomitant chronic diseases. For both adherence and persistence, male sex was significantly associated with higher adherence and higher persistence. However, contradictory results have been found in the literature. A study by Kane et al. showed that males were twice as likely as females to be non-adherent with their UC treatment [23]. This discrepancy in results could be due to differences in patient population for 2 studies; the Kane et al. study was conducted with small number (n = 94) of US patients at the University of Chicago, whereas this study is with Canadian patients. Also patients in the Kane et al. study were younger than patients in the current study (51 vs. 55 years old). However, another study by Kane et al. demonstrated that females decreased persistence at 3 months [27] while Ediger et al. showed that younger females were less adherent than older males [29]. This latter study also reported that predictors of adherence differed markedly between the sexes. These results demonstrated the complexity of adherence in UC, which seemed to differ between individuals. This complexity was also observed in presence of concomitant chronic diseases, which have a stronger impact on adherence than persistence [30,31]. Although some of the covariates in our study have a significant impact on adherence and persistence, this impact is somewhat limited with ORs of 1.2 to 1.6.

As this study was performed using data from an administrative claims database, it enables the inclusion of a large number of subjects that are assumed to accurately represent the general population of Quebec province of Canada and in a real-world clinical setting, contrary to a clinical trial, with relatively small sample size and controlled clinical environment. Specifically in contrast to this study, clinical trials reported excellent rates of adherence with 5-ASA therapy, in the order of 80% of patients or more [32,33]. As participants are generally better motivated and under close medical supervision compared with those receiving therapy outside the clinical trial setting, these rates do not necessarily reflect real-world use and from this point of view, the RAMQ database allows a more accurate estimation of adherence and persistence. However, as in other studies based on administrative databases, there are some inherent limitations. It is assumed that reimbursed medications retrieved from the databases were taken by the patient, although this may not be always the case. Also, drug samples received by the patient from his/her physician, medications received while hospitalized and medication not reimbursed by the drug plan, including over-the-counter medications, are not taken into account. Another limitation of this study was the relatively older age of the patient population who were covered under a free drug prescription benefit. Moreover, the presence of a claim for a filled prescription does not necessarily indicate that the medication was consumed or that it was taken as prescribed, which can potentially affect adherence results. Furthermore, *some patients with UC may have been excluded if they also carry a diagnosis of CD. Also, some patients with CD may have been kept if they were never given a CD diagnosis. This limitation is inherent to this kind of database, but is also a reflection of the difficulties of distinguishing betwenn these two diagnoses in clinical practice. Neverthess, this would have had a limited impact on the relative adherence and persistence of one medication compared to another.*

## Conclusions

In summary, this RAMQ-based analysis provides descriptive information regarding prescription characteristics and 5-ASA adherence and persistence. The overall findings demonstrated that, in a real-life setting, adherence and persistence were poor in 5-ASA patients, although it was improved with Mezavant®, suggesting that once daily treatments could help to improve adherence and persistence of 5-ASA patients and may lead to a decrease in frequency of UC exacerbations. Reasons for adherence and persistence to 5-ASA treatments are complex and vary from 1 patient to another. Better understanding of determinants may improve UC management by providing strategies to improve adherence and persistence and thereby reducing relapses.

## Abbreviations

5-ASA: 5-aminosalicylic acid; ANOVA: One-way analysis of variance; CI: Confidence interval; MPR: Medication possession ratio; RAMQ: Régie de l’assurance maladie du Québec; OR: Odds ratio; UC: Ulcerative colitis.

## Competing interests

Jean Lachaine and Catherine Beauchemin, disclose receipt of funding as independent contractors from Shire Pharmaceuticals LLC. Linnette Yen and Paul Hodgkins are employed by Shire Pharmaceuticals.

## Authors’ contributions

JL, CB, LY and PH participated in the design of the study. JL and CB performed the analyses and drafted the manuscript. LY and PH revised the manuscript. All authors read and approved the final manuscript.

## Authors’ information

JL, PhD, is associate professor in pharmacoeconomics at the Faculty of Pharmacy, University of Montreal. LY, MS, MA, is director of Global Health Economics and Outcomes Research at Shire Development. CB, MSc, is PhD student in pharmacoeconomics at the Faculty of Pharmacy, University of Montreal. PH, PhD, is senior director of Global Health Economics and Outcomes Research at Shire Development.

## Pre-publication history

The pre-publication history for this paper can be accessed here:

http://www.biomedcentral.com/1471-230X/13/23/prepub
